# A new test for autism spectrum disorder: Metabolic data from different cell types

**DOI:** 10.1016/j.dib.2021.107598

**Published:** 2021-11-23

**Authors:** Sujata Srikanth, Lauren Cascio, Rini Pauly, Kelly Jones, Skylar Sorrow, Rossana Cubillan, Chin-Fu Chen, Cindy D. Skinner, Kevin Champaigne, Roger E. Stevenson, Charles E. Schwartz, Luigi Boccuto

**Affiliations:** a Greenwood Genetic Center, 101 Gregor Mendel Circle, Greenwood, SC 29646, USA; b Circa Bioscience, Greenwood, 113 Gregor Mendel Circle, Greenwood, SC 29646, USA; c School of Nursing, College of Behavioral, Social and Health Sciences, Clemson University, 508 Edwards Hall, Clemson, SC 29634, USA

**Keywords:** Autism spectrum disorder (ASD), Tryptophan, Metabolism, Diagnostic test, Screening test

## Abstract

Experiments employing the Phenotype Mammalian Microarray (PM-M) technology were performed on lymphoblastoid cell lines (LCLs) from individuals with autism spectrum disorder (ASD) and age-matched controls. We used the custom-made PM-M plate designed to assess differential utilization of the amino acid tryptophan. Multiple parameters such as the sample size, incubation time, and cell concentration have been tested, leading to optimized protocols and minimized background noise by variable selection while controlling for false discoveries. The assay generated data based on the production of nicotinamide adenine dinucleotide (NADH) in the presence of different compounds containing tryptophan and showed clear differences between ASD and control samples.

## Specifications Table

**Table:** 

Subject	Health and Medical Sciences
Specific subject area	Metabolic assay for autism spectrum disorder
Type of data	Table Figure
How data were acquired	Biolog OmniLog, serial number 455, model 270001
Data format	Raw Analyzed
Parameters for data collection	Different cell concentrations per well, incubation time and amount of fetal bovine serum were considered for data collection.
Description of data collection	Data were collected after different times of incubation via optical density and endpoint absorbance readings. In the “feeding schedule” Supplemental file we reported three sets of data (endpoint absorbance, endpoint optical density, and slope for optical density curve over 24 h) collected from experiments with different schedules for cell feeding and plating. In the “Cell concentration-FBS combinations” Supplemental file we reported three sets of data (endpoint absorbance, endpoint optical density, and slope for optical density curve) with their respective averages, collected from experiments with different combinations of cell concentrations per well (10,000, 20,000, and 40,000), FBS concentration (1% and 5%), and incubation times (24, 48, and 72 h). In the “normalized 50v50 ABS average (old vs. new protocol)” Supplemental file we reported normalized endpoint absorbance data for 50 ASD and 50 control lymphoblastoid cell lines for two protocols: 48 h of incubation, 5% FBS, and 20,000 cells/well (the original protocol) versus 72 h of incubation, 1% FBS, and 40,000 cells/well (the modified protocol).
Data source location	Institution: Greenwood Genetic Center City: Greenwood, SC Country: USA Latitude and longitude for collected samples/data: 34.1954° N, 82.1618° W
Data accessibility	Boccuto, Luigi (2021), “Biolog data for ASD”, Mendeley Data, V1, https://doi.org/10.17632/6z6jyxzwhd.1
Related research article	R. Pauly, L. Cascio, S. Srikanth, K. Jones, S. Sorrow, R. Cubillan, C.F. Chen, C. D. Skinner, K. Champaigne, R. E. Stevenson, C. E. Schwartz, L. Boccuto, Development of a cell-based metabolic test for the identification of individuals with autism spectrum disorder, Res. Autism Spectr. Disord. 85 (2021) https://doi.org/10.1016/j.rasd.2021.101790

## Value of the Data

• The data we generated provide important validation of a novel protocol for a cell-based metabolic assay that may lead to a diagnostic test for autism spectrum disorder (ASD). The study explored modifications of multiple parameters in order to select the best protocol for the tested arrays.
• The clinical diagnosis of ASD is still challenging, occurs on average around the third year of age, and can only rely on behavioral observation. The data we report can pave the way for a cell-based test representing an objective tool for validating the diagnosis and identifying individuals at risk of developing ASD even before the onset of the behavioral symptoms. Therefore, families and physicians can benefit from an earlier and more accurate diagnosis of ASD.
• The data reported in this paper represents the first validation of a new and improved protocol for a cell-based metabolic test for ASD. They can be used for future studies on larger ASD cohorts, for investigating potential metabolic profiles associated with ASD comorbidities, and for developing novel therapeutic approaches.

## 1 Data Description

The experiments are designed to measure the production of nicotinamide adenine dinucleotide (NADH) in the presence of tryptophan in both ASD and TD lymphoblastoid cells. The ASD cells were obtained from individuals diagnosed according to the DSM-IV-TR guidelines, utilizing the Autism Diagnostic Observation Schedule (ADOS) diagnostic instrument. The TD cells were obtained from age-matched individuals evaluated for neurobehavioral development. Further information on the tested cohort is reported in the related research article and in a previous paper [1].

The original protocol, developed by Biolog, requires a 5% concentration of fetal bovine serum (FBS) in the media, 4 × 10^5^ cells/mL (20,000 cells/well), and 48 h of incubation before the addition of the dye. The effects of the modification of various parameters were tested both individually and as part of combined protocols.

### 1.1 Feeding schedule (Supplemental file)

The feeding schedule of the lymphoblastoid cell lines (LCLs) before plating on the PM-M arrays was examined to assess its potential effect on the cells’ performance on the Biolog plates, four feeding schedules were tested on cell lines from 4 individuals with ASD and 4 typically developing (TD) individuals. The first schedule tested was the original protocol. The cells were harvested and plated on Biolog plates 72 h after they were fed RPMI media (growing media). Dye was then added 48 h after the cells were plated. For the second schedule tested the only difference was that the cells were fed Biolog media instead of growing media 72 h before being harvested and plated. For the third schedule, the cells were fed Biolog media in the flask 24 h before being plated. For the final schedule, the cells were fed with Biolog media containing no serum 24 h before being plated.

### 1.2 Cell concentration-FBS combinations (Supplemental file)

The plating step was examined next in order to determine the impact of this step. Several components of this step could have potentially affected the cells’ performance, including FBS type and concentration, cell concentration, and incubation time.

FBS Concentration. The file contains individual and average data from 2 ASD and 2 TD samples for endpoint absorbance, endpoint optical density, and slope. The first parameter of the original protocol that was altered was the FBS. The same FBS used for cell culturing was tested at concentrations of 5, 10, 15, and 20%.

Cell concentration. The data reported were collected from experiments assessing the NADH production with concentrations of 8 × 10^5^ cells/mL (40,000 cells/well), 12 × 10^5^ cells/mL (60,000 cells/well), and 16 × 10^5^ cells/mL (80,000 cells/well).

Incubation time. A cohort of 10 ASD and 10 TD samples was used to assess three incubation times preceding the addition of dye: 0, 24, and 48 h. For the 0-h incubation, the cells were plated in Biolog media and the dye was added simultaneously, then the plates were placed in the Omnilog for 24 h. For the 24-h incubation, the cells were plated in media and incubated for 24 h, then the dye was added and the plate was placed in the Omnilog for 24 h.

Incubation of 72 h was also tested using a cohort of 2 ASD and 2 TD LCLs. The cells were plated in media containing 1%, 5%, and 10% FBS. After 72 h, the dye was added and the plate was placed in the Omnilog for 24 h.

Combination. Based on the data generated from the experiments described above, the top candidates for each parameter along with additional candidates were then combined and tested on a cohort of 2 patients with ASD and 2 TD individuals: individual and average data for endpoint absorbance, endpoint optical density, and slope are listed in the file.

The following combinations were tested with both 0 and 24 h of incubation: 10,000, 20,000, and 40,000 cells/well (2, 4, and 8 × 10^5^ cells/mL) in 1% FBS media; 10,000 and 40,000 cells/well (2 and 8 × 10^5^ cells/mL) in 5% FBS media.

The following combinations were tested with both 48 and 72 h of incubation: 10,000, 20,000, and 40,000 cells/well (2, 4, and 8 × 10^5^ cells/mL) in both 1% and 5% FBS media.

### 1.3 Old vs. New 50ASDvs50Controls (Supplemental file)

**Fig. 1:**
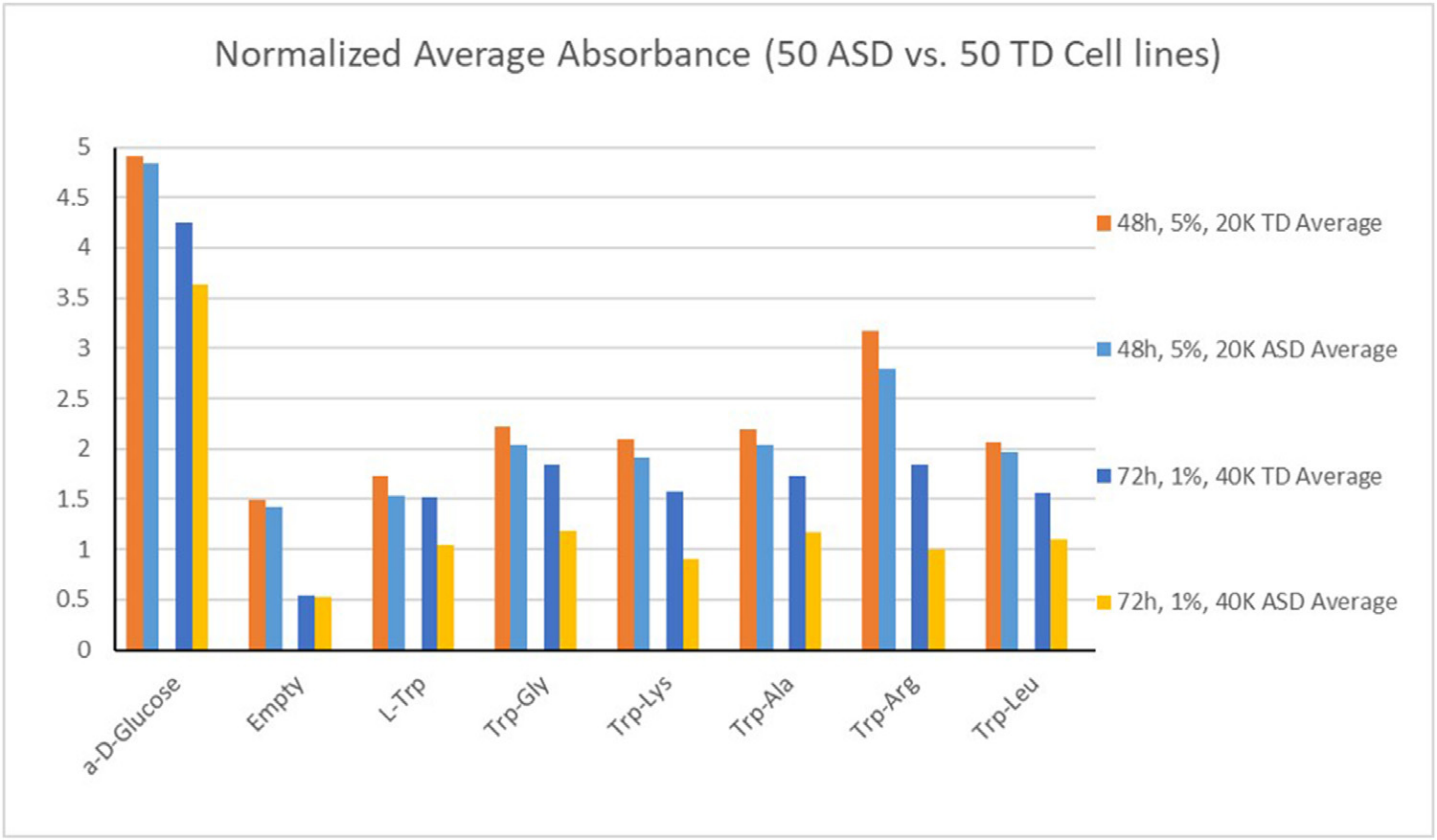
The figure represents graphically the data reported in the “Old vs. New 50ASDvs50Controls” file: the average endpoint absorbance values for the 50 ASD and 50 TD LCLs tested on the new protocol – 40,000 cells/well, 1% FBS, 72-h incubation – as compared to the original one –20,000 cells/well, 5.% FBS, 72-h incubation.

In consideration of all the experiments performed, the best overall candidate protocol for LCLs resulted to be the one with 8 × 10^5^ cells/mL (40,000 cells/well) in 1% FBS and 72 h of pre-dye incubation. This protocol was compared versus the original one: 4 × 10^5^ cells/mL (20,000 cells/well) in 5% FBS and 48 h of pre-dye incubation. The two protocols were tested on 50 ASD and 50 TD LCLs Fig. 1.

## 2 Experimental Design, Materials and Methods

The experiments were designed to assess the capacity of the custom-made PM-M Trp assay to distinguish the metabolic profile of ASD samples from the one of typically developing (TD) individuals. The tested parameters include:

- fetal bovine serum (FBS) concentrations: 5%, 10%, 15% and 20%.
- incubation time: 0, 24, and 48 h.
- cell concentration: 4 × 10^5^ cells/mL (20,000 cells/well), 8 × 10^5^ cells/mL (40,000 cells/well), 12 × 10^5^ cells/mL (60,000 cells/well), and 16 × 10^5^ cells/mL (80,000 cells/well).

### 2.1 ASD and TD cell lines

Experiments were performed utilizing cell lines generated from 50 children with non-syndromal ASD and 50 age-matched TD controls from the same geographic area. Overall, 43 individuals with ASD were male and 7 were female (6.1:1 male-to-female ratio), with an age range of 1 to 11.1 years (average 4.94 years) at the time of blood sample acquisition, and they were living in the states of South and North Carolina. TD individuals ranged in age from 11 months to 11.3 years (average 5.42 years) at the time of blood sample acquisition and were living in the same geographic area. For both ASD and TD cohorts, peripheral blood samples were collected by venipuncture and then lymphocytes were isolated via centrifugation. LCLs were obtained by immortalization via Epstein-Barr virus of lymphocytes isolated from the blood samples. The LCLs were cultured in Sigma RPMI-1640 with 15% fetal bovine serum (FBS) from Atlanta Biological (Flowery Branch, GA, USA) and 2 mM L-Glutamine, 100 U/ml Penicillin and 100 µg/ml Streptomycin from Sigma-Aldrich (St. Louis, MO, USA). Only cell lines with a viability of 55% or greater as measured by an automated cell counter were used for the experiments.

### 2.2 Biolog metabolic arrays

The protocol initially used for this plate was developed by the Biolog (Hayward, CA, USA) for their Phenotype Mammalian Microarray (PM-M) plates, specifically, the ones designed to measure the production of nicotinamide adenine dinucleotide (NADH) from carbon energy sources (PM-M1 to M4) [2]. This original protocol has been described in detail in previous studies [1], [2], [3]. In this project, we utilized a custom-made PM-M plate constituted of twelve 8-well columns containing α-D-glucose (positive control), an empty well (internal negative control), L-tryptophan (Trp), and five tryptophan dipeptides (Trp-Gly, Trp-Lys, Trp-Ala, Trp-Arg, and Trp-Leu). All experiments were conducted in triplicates. The cells were incubated for 48 h at 37°C in 5% CO_2_, after which Biolog Redox Dye Mix MB was added (10 μL/well) and the plates were incubated in the Omnilog system under the same conditions for an additional 24 h. As the cells metabolize the energy source provided in the well, tetrazolium dye in the media is reduced, producing a purple color according to the amount of NADH generated. At the end of the 24-h incubation, the plates were analyzed utilizing a microplate reader with readings at 590 and 750 nm. The first value (A_590_) indicated the highest absorbance peak of the redox dye and the second value (A_750_) gave a measure of the background level. The relative absorbance (A_590-750_) was calculated per well.

### 2.3 Statistical analysis

Using Biolog PM-M arrays we collected data on multiple metabolic pathways, utilizing both endpoint absorbance and kinetic readings of optical density over 24 h of incubation. The data is normalized using a triplicate of empty plates, containing only the Biolog medium without cells. Then the absorbance readings were transformed to a logarithmic scale to compare the metabolic profiles of individuals with ASDs to that of TD controls. We utilized the program RStudio (version 1.1.456) to implement the non-parametric Mann-Whitney's *t*-test to determine differences in terms of significant wells between the ASD and TD samples. False discovery rates were controlled for multiple testing using the Benjamini and Hochberg technique of *p*-value correction. The wells are considered significant if the adjusted *p*-value is less than 0.05.

## Ethics Statement

Informed consent forms were reviewed and signed by all the participants evaluated and/or their legal guardians. The consent forms and research protocol were approved by the Self Regional Healthcare Institutional Review Board (IRB) for Human Research.

## CRediT authorship contribution statement

**Sujata Srikanth:** Data curation, Writing – original draft. **Lauren Cascio:** Data curation, Writing – original draft. **Rini Pauly:** Formal analysis. **Kelly Jones:** Data curation, Writing – original draft. **Skylar Sorrow:** Data curation, Writing – original draft. **Rossana Cubillan:** Data curation, Writing – original draft. **Chin-Fu Chen:** Formal analysis. **Cindy D. Skinner:** Resources. **Kevin Champaigne:** Methodology. **Roger E. Stevenson:** Conceptualization, Writing – review & editing. **Charles E. Schwartz:** Conceptualization, Writing – review & editing. **Luigi Boccuto:** Conceptualization, Methodology, Supervision, Writing – review & editing.

## Declaration of Competing Interest

The authors declare that they have no known competing financial interests or personal relationships which have or could be perceived to have influenced the work reported in this article.

## Appendix Supplementary materials

Image, application 1

Image, application 2

Image, application 3
